# Medicare Costs by Disability and Community-Dwelling Status

**DOI:** 10.1093/geroni/igab046.450

**Published:** 2021-12-17

**Authors:** Claire Ankuda, Katherine Ornstein

**Affiliations:** Icahn School of Medicine at Mount Sinai, New York, New York, United States

## Abstract

Understanding population-level Medicare expenditure patterns for older adults with functional disability is critical to focus supports to reduce costly and potentially burdensome health care use. We used the National Health and Aging Trends Study (NHATS) to assess quarterly Medicare expenditures over the 12 months following NHATS interview. We examine Medicare expenditure patterns for older adults in nursing homes (N=386), in the community and without disability (N=20,103), with disability and dementia (N=2,008), and with disability but not dementia (N=2,945). One-year mortality ranged from 2.0% for those without disability in the community to 25.9% for those residing in nursing homes. Among those surviving 1 year, Medicare expenditures the first quarter after NHATS survey ranged from $1,794 (95% CI $1,690-$1,898) for those with no disability to $5,177 (95% CI $4,535-$5,818) for those with disability and dementia. We assess trends over the following two years, and find that trajectories vary by clinical grouping.

